# Detrimental Effects of Chronic L-Arginine Rich Food on Aging Kidney

**DOI:** 10.3389/fphar.2020.582155

**Published:** 2021-01-19

**Authors:** Ji Huang, Diogo Ladeiras, Yi Yu, Xiu-Fen Ming, Zhihong Yang

**Affiliations:** ^1^ Department of Endocrinology, Metabolism, and Cardiovascular System, Laboratory of Cardiovascular and Aging Research, Faculty of Science and Medicine, University of Fribourg, Fribourg, Switzerland; ^2^ National Center of Competence in Research “Kidney.CH”, University of Zürich, Zürich, Switzerland

**Keywords:** aging, arginase, kidney, arginine supplementation, inflammation

## Abstract

The impaired L-arginine/nitric oxide pathway is a well-recognized mechanism for cardiovascular and renal diseases with aging. Therefore, supplementation of L-arginine is widely proposed to boost health or as adjunct therapy for the patients. However, clinical data, show adverse effects and even enhanced mortality in patients receiving long-term L-arginine supplementation. The effects of long-term L-arginine supplementation on kidney aging and the underlying mechanisms remain elusive. Moreover, high protein and high amino acid diet has been thought detrimental for kidney. We therefore investigated effects of chronic dietary L-arginine supplementation on kidney aging. In both young (4 months) and old (18–24 months) mice, animals either receive standard chow containing 0.65% L-arginine or diet supplemented with L-arginine to 2.46% for 16 weeks. Inflammation and fibrosis markers and albuminuria are then analyzed. Age-associated increases in *tnf-α*, *il-1β*, *and il-6*, *vcam-1*, *icam-1*, *mcp1*, *inos*, and macrophage infiltration, collagen expression, and S6K1 activation are observed, which is not favorably affected, but rather further enhanced, by L-arginine supplementation. Importantly, L-arginine supplementation further enhances age-associated albuminuria and mortality particularly in females, accompanied by elevated renal arginase-II (Arg-II) levels. The enhanced albuminuria by L-arginine supplementation in aging is not protected in Arg-II^−/−^ mice. In contrast, L-arginine supplementation increases ROS and decreases nitric oxide production in old mouse aortas, which is reduced in Arg-II^−/−^ mice. The results do not support benefits of long-term L-arginine supplementation. It rather accelerates functional decline of kidney and vasculature in aging. Thus, the long-term dietary L-arginine supplementation should be avoided particularly in elderly population.

## Introduction

L-arginine is a semi-essential amino acid involved in protein synthesis and is the substrate for nitric oxide synthase (NOS) to produce the vascular protective nitric oxide (NO) released from the endothelial cells (Wu et al., 2009). Since decreased bioavailability of NO or deficiency of NO production promotes development of cardiovascular diseases and chronic kidney diseases, and is highly associated with aging (Schmitt and Melk, 2017; Donato et al., 2018), supplementation of L-arginine has been proposed to increase endothelial NO bioavailability and to improve health status in the young as well as in elderly population or as an adjunct therapeutic modality to treat patients with cardiovascular diseases (Creager et al., 1992). However, mixed results on the therapeutic effects of L-arginine supplementation in either experimental models or clinical studies are reported and there is continuous debate on whether L-arginine shall be supplemented or shall be avoided and in which context of diseases (Dioguardi, 2011; Nogiec and Kasif, 2013; Hadi et al., 2019; Nitz et al., 2019). While some studies demonstrate improvement of cardiovascular functions or reduced risks (Rector et al., 1996; Maxwell et al., 2000; Dong et al., 2011), numerous other studies with long term L-arginine supplementation showed either no sustained effects (Sato et al., 2000; Meirelles and Matsuura, 2018; Rodrigues-Krause et al., 2018) or harmful effects and even increased mortality in patients with cardiovascular disease (Chen et al., 2003; Schulman et al., 2006; Wilson et al., 2007). A recent Mendelian randomization study proposed that high L-arginine levels are associated with higher risk of ischemic heart disease (Au Yeung et al., 2016) which further indicates that chronic L-arginine supplementation may cause harmful effects. The underlying mechanisms remain elusive.

Kidneys are important organs participating in amino acid metabolism including L-arginine (Makrides et al., 2014). Kidneys express all the enzymes in the L-arginine metabolism, including NOS and arginase, particularly arginase-II (Arg-II) (Huang et al., 2016), the enzyme Arg-II metabolizes L-arginine to urea and L-ornithine (Moretto et al., 2019). With development of chronic kidney disease, L-arginine deficiency may occur and L-arginine supplementation as an approach to restore L-arginine bioavailability to treat kidney disease has been proposed (Baylis, 2006). Similar to the cardiovascular system, the effects of dietary L-arginine supplementation on kidney function are also controversial (Tome et al., 1999). Both renoprotective effects (Korish, 2010; Carlstrom et al., 2013) and lack of the beneficial effects (You et al., 2014) or detrimental effects have been reported (Peters et al., 2003).

Aging is an important risk factor of chronic kidney disease (O’Sullivan et al., 2017) and is accompanied by global glomerulosclerosis, tubulointerstitial fibrosis, and chronic inflammation (Hommos et al., 2017). In the cardiovascular system, a short term supplementation of L-arginine increases endothelial NO production, while chronic L-arginine supplementation stimulates endothelial cell senescence and decreases endothelial function linked to sustained activation of mTORC1 pathway and arginase type II (Arg-II) (Xiong et al., 2014). The safety and efficiency of prolonged use of L-arginine supplementation in the kidney, particularly in old age, is not clear. Taken into account that high protein and high amino acid diet is a risk factor for renal function (Friedman, 2004), the present study is aimed to clarify the effects of chronic dietary L-arginine supplementation in the kidney of young and old mice of males and females and to investigate whether this is related to Arg-II, the enzyme that is highly and constitutively expressed in kidneys (Huang et al., 2016).

## Results

### Effects of Chronic L-Arginine Supplementation on Age-Associated Renal Inflammation and Collagen Gene Expression

**FIGURE 1:**
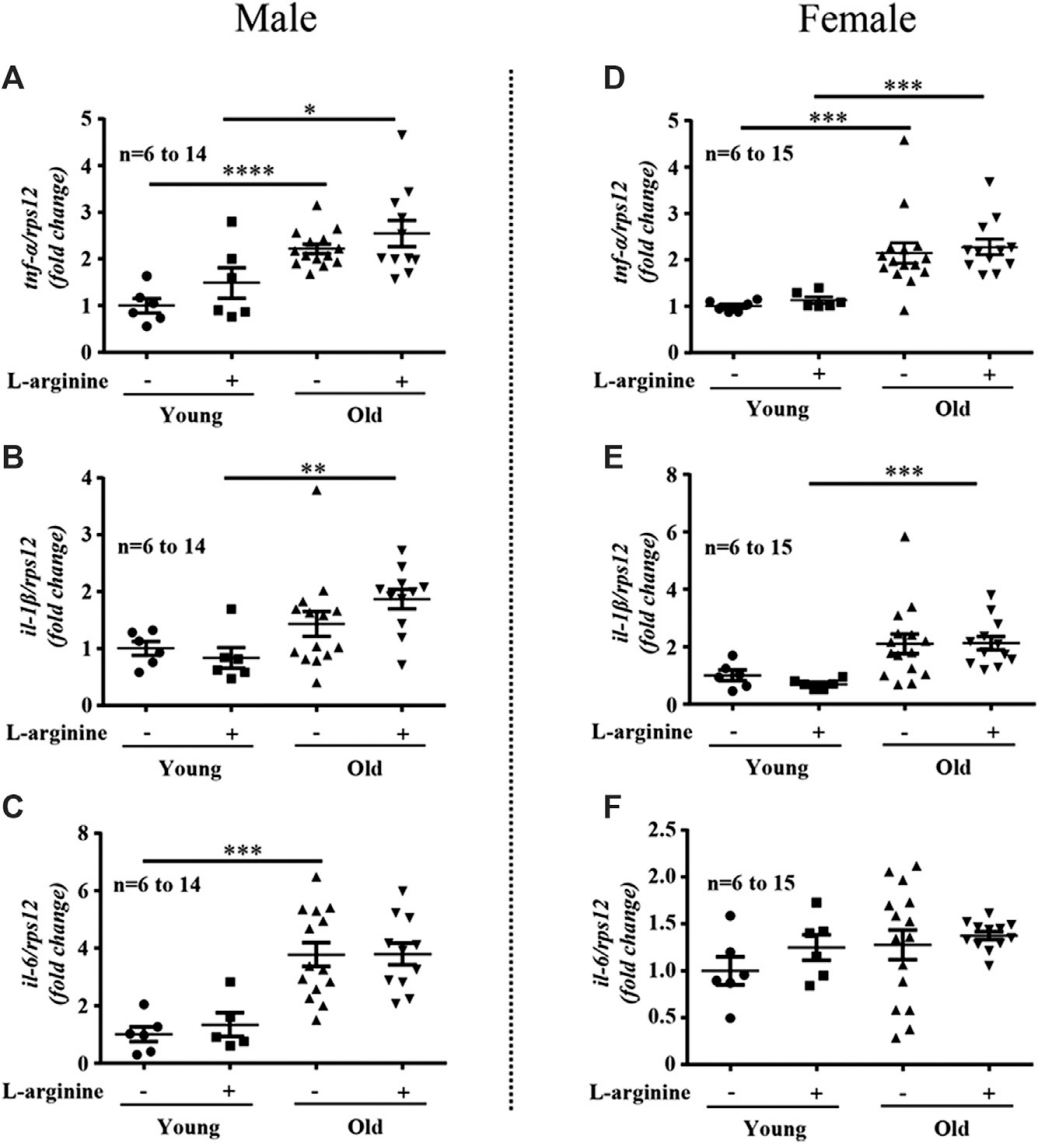
L-arginine supplementation does not mediate inflammatory cytokine production. Renal mRNA expression of pro-inflammatory cytokines *tnf-α*, *il-1β*, and *il-6* were measured in whole kidney lysate of male **(A–C)** and female **(D–F)** mice. *rps12* served as the reference. The values shown are mean ± SEM. *n* indicates the number of animals used in the experimental groups. Data is expressed as the fold change to the young control group. *p ≤ 0.05, **p ≤ 0.01, ***p ≤ 0.001, ****p ≤ 0.0001.

**FIGURE 2:**
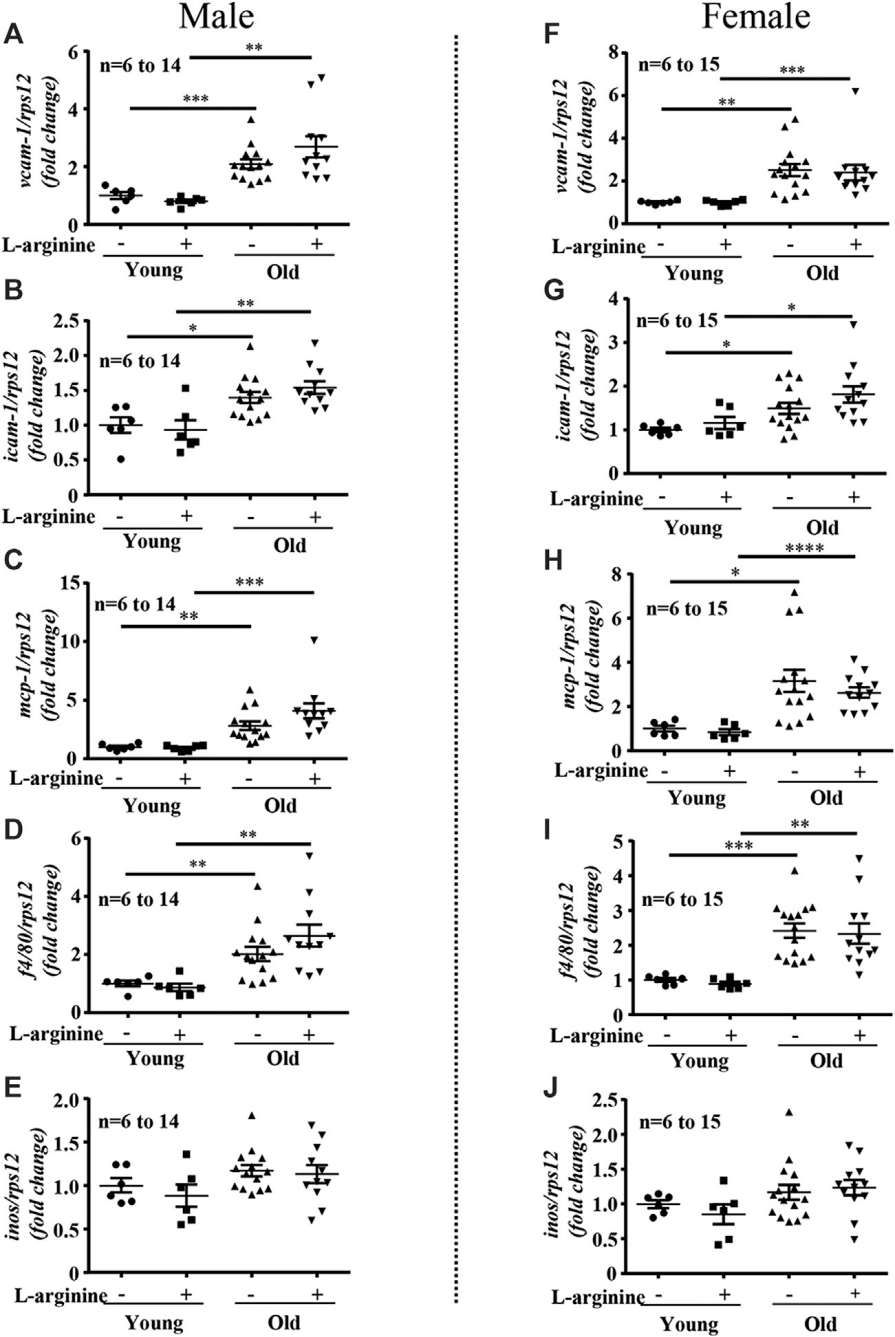
L-arginine supplementation does not mediate renal macrophage infiltration. mRNA expression of adhesion molecules *vcam-1* and *icam-1* were evaluated by qRT-PCR in whole kidney lysate of male **(A,B)** and female **(F,G)** mice. *mcp-1*, macrophages marker *f4/80* and *inos* gene expression were assessed in kidney of young and old male **(C–E)** and female **(H–J)** mice. *rps12* served as the reference. The values shown are mean ± SEM. *n* indicates the number of animals used in the experimental groups. Data is expressed as the fold change to the young control group. *p ≤ 0.05, **p ≤ 0.01, ***p ≤ 0.001, ****p ≤ 0.0001.

**FIGURE 3:**
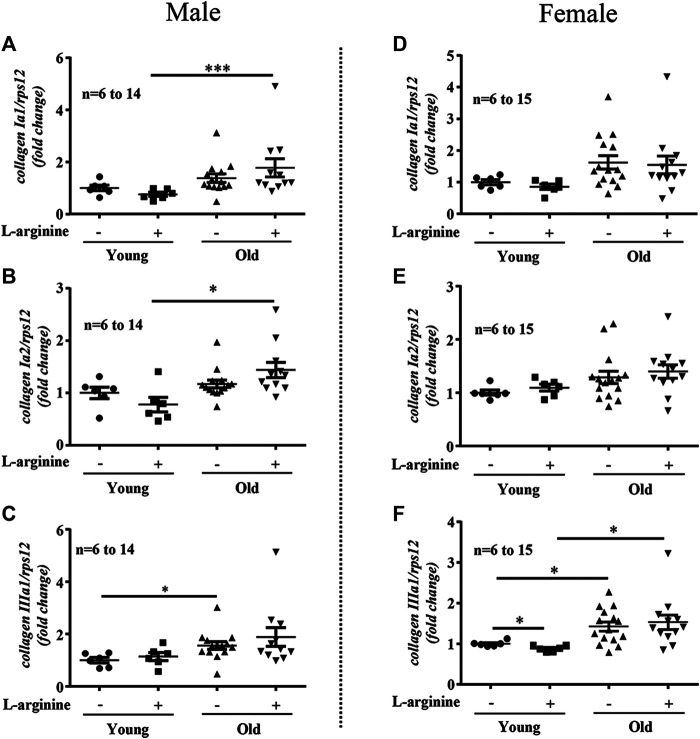
L-arginine supplementation does not influence renal collagens expression. mRNA analysis of *collagen Iα1*, *collagen Iα2* and *collagen IIIα1* through qRT-PCR in whole kidney of control and L-arginine supplemented male **(A–C)** and female **(D–F)** mice. *rps12* served as the reference. The values shown are mean ± SEM. *n* indicates the number of animals used in the experimental groups. Data is expressed as the fold change to the young control group. *p ≤ 0.05, ***p ≤ 0.001.

There was an age-associated increased expression of numerous inflammatory cytokines/molecules, including *tnf-α*, *il-1β*, and *il-6*, *vcam-1*, *icam-1*, *mcp1*, *inos*, and macrophage marker *f4/80* in the kidney in male and female mice (Figures 1, 2). L-arginine supplementation for 4 months had no or only negligible effects on these parameters either in young or old mice of both males and females (Figures 1, 2). Moreover, there was also an age-associated increase in renal *collagen Iα1*, *collagen Iα2*, and particularly *collagen IIIα1* in both males and females, which was however, not significantly affected by chronic L-arginine supplementation (Figure 3).

### Effects of Chronic L-Arginine Supplementation on Aging Marker p16 ^INK4a^, Arg-II, and S6K1 Activation

**FIGURE 4:**
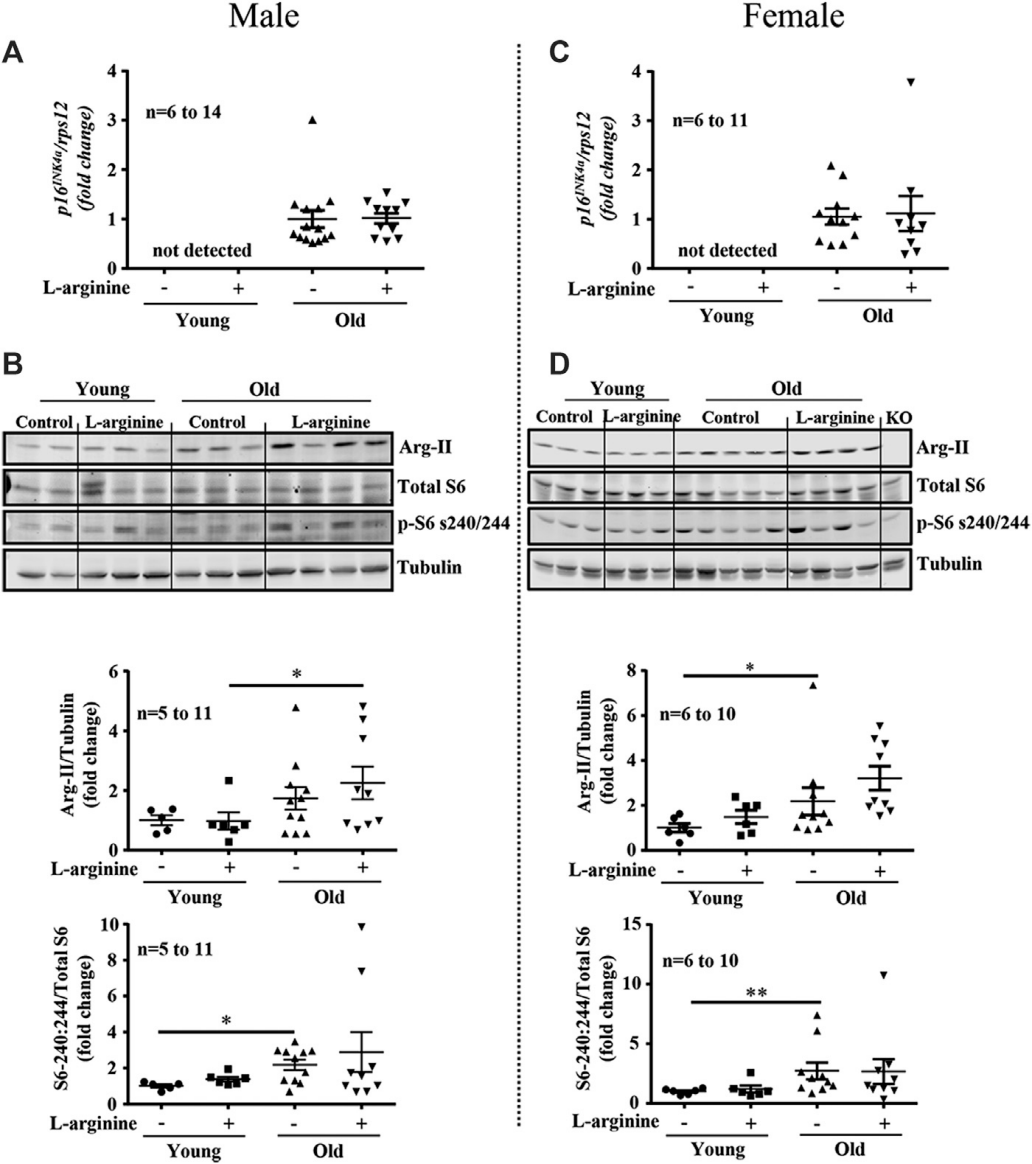
Prolonged L-arginine diet does not affect *p16* ^*INK4a*^, Arg-II or S6K1 pathways. Aging marker *p16* ^*INK4a*^ mRNA expression was evaluated in male **(A)** and female **(C)** kidney of control and L-arginine supplemented mice. *rps12* was used as reference gene. Immunoblotting analysis of Arg-II and S6 protein (S6-S240/244) and total S6 in kidney lysates of control and supplemented male **(B)** and female **(D)** mice. n indicates the number of animals used in the experimental groups. Dot plots show quantifications of the markers. Tubulin was used as loading control. The values shown are mean ± SEM. *p ≤ 0.05, **p ≤ 0.01.

Since L-arginine is an strong activator of mTORC1/S6K1 signaling pathway which is involved in accelerating cellular senescence and organism aging, we have analysed whether L-arginine supplementation could activate this signaling pathway and accelerate renal aging. Increased expression of aging marker *p16* ^*INK4a*^ was demonstrated in old male and female mice as compared to the young mice (Figures 4A,C), which was confirmed by immunofluorescence staining in cortex and medulla analysed by confocal microscopy (Supplementary Figure S1). Chronic L-arginine supplementation did not affect the aging marker as demonstrated either by qRT-PCR (Figures 4A,C) or immunofluorescence staining (Supplementary Figure S1). Also an increase in mTORC1/S6K1 signaling as measured by the enhanced ratio of phosphor-S240:244-S6/total S6 was observed in the old mice. This was however, not influenced by chronic L-arginine supplementation (Figures 4B,D). An age- associated increase in arginase-II (Arg-II) was found in the mice, which tended to be increased with L-arginine supplementation, particularly in females (Figures 4B,D).

### Effects of Chronic L-Arginine Supplementation on Albuminuria in Aging

**FIGURE 5:**
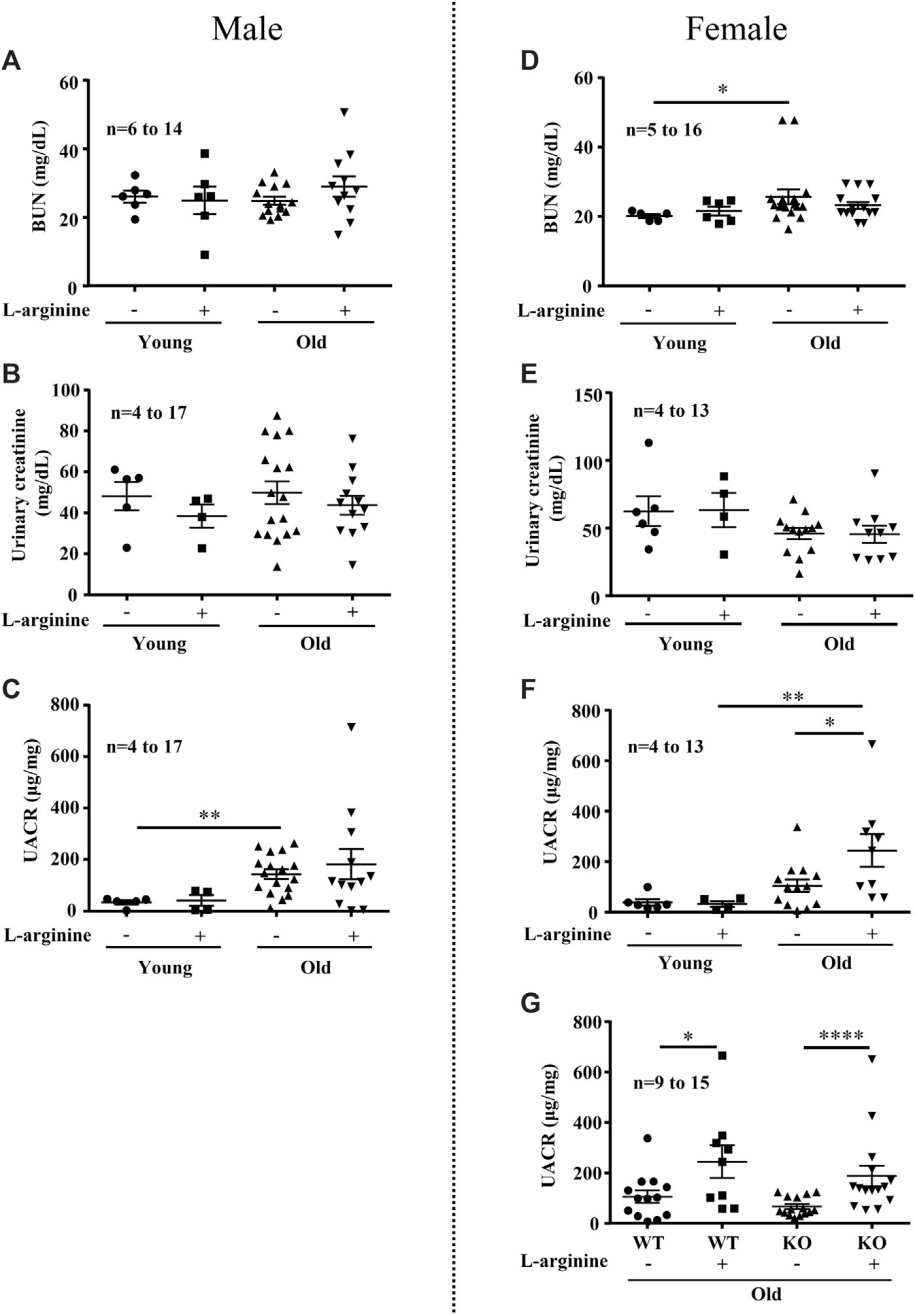
Long-term L-arginine supplementation induced albuminuria in aged females. Renal functional markers blood urea nitrogen (BUN, mg/dl), urine creatinine and albuminuria (UACR) were accessed in male **(A–C)** and female **(D–F)** mice under control and L-arginine diet. UACR was evaluated in aged WT and Arg-II^−/−^ female mice under L-arginine food **(G)**. *n* indicates the number of animals used in the experimental groups. The values shown are mean ± SEM. *p ≤ 0.05, **p ≤ 0.01, ****p ≤ 0.0001.

We further analyzed the effects of chronic L-arginine supplementation on urinary albumin-creatinine ratio (UACR) and blood urea nitrogen (BUN) as kidney functional biomarkers. There was a significant increase in BUN levels in the female (not male) mice with aging (Figures 5A,D). No difference in urinary creatinine was observed among the groups (Figures 5B,E). An age-associated increase in UACR was observed in both male and female mice (Figures 5C,F). Remarkably, chronic L-arginine supplementation significantly enhanced the aging-associated increase in UACR in the old female mice (Figure 5F). This increase in UACR induced by chronic L-arginine supplementation in the old mice remained unaffected in mice with Arg-II deficiency (Arg-II^−/−^) (Figure 5G). The results indicate that L-arginine induced UACR in aging is independent on arginase-II.

### Effects of Chronic L-Arginine Supplementation on Mortality in Aged Mice

**FIGURE 6:**
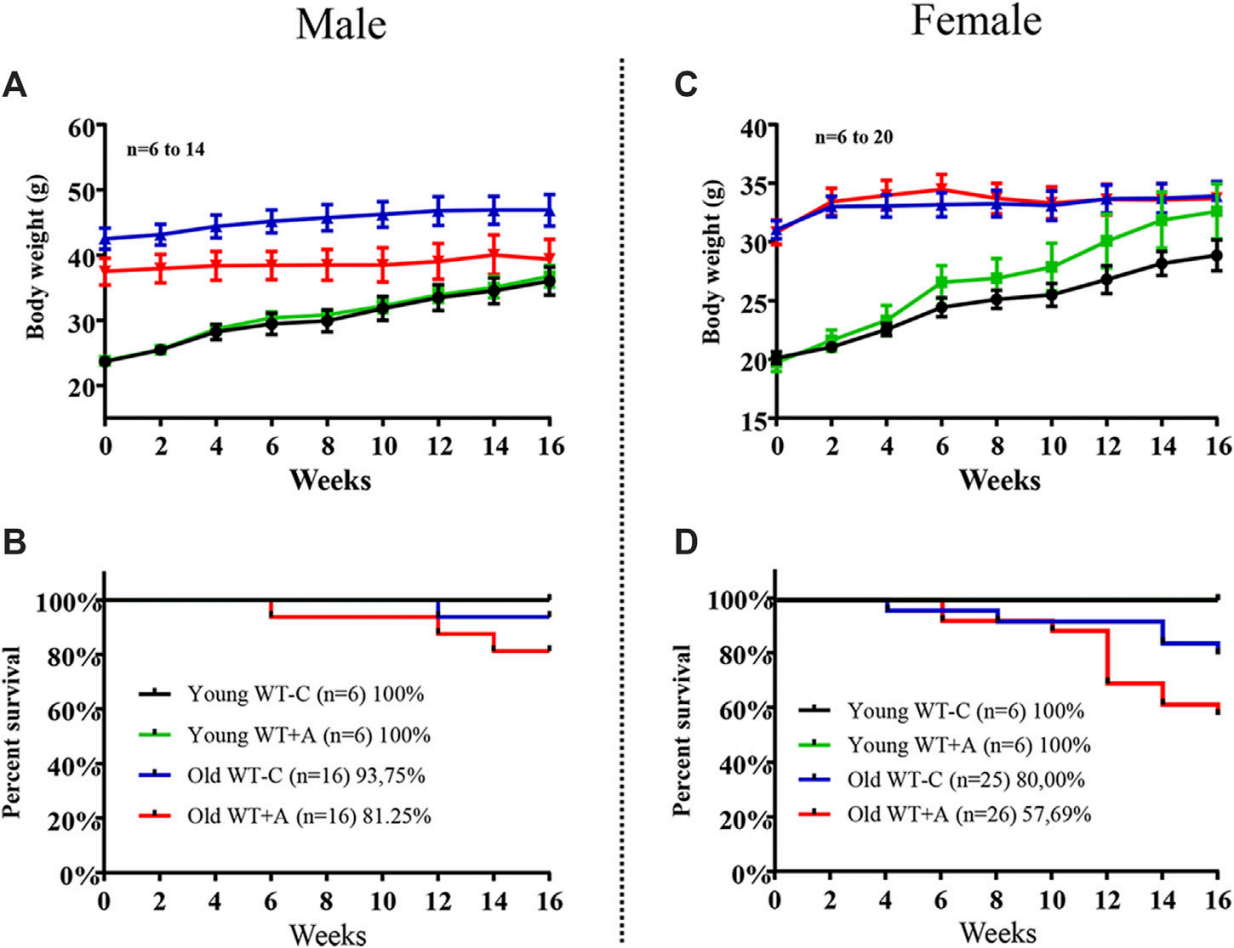
Long-term L-arginine supplementation increases mortality rate in aged mice under supplementation. The body weight and the number of animals under control diet and L-arginine supplementation were monitored every 2-weeks during the 16-weeks period of dietary L-arginine treatment in male **(A,B)** and female **(C,D)** young and old mice. *n* indicates the number of animals in the experiment.

Old mice, both males and females, revealed no alteration in body weight during the 16-week period of L-arginine supplementation. As expected, young mice gained weight over time in a faster rate in female mice under L-arginine supplementation as compared with mice under control diet, although it is statistically not significant (Figures 6A,C). It is of interest to note that the mortality rate of old mice was increased by chronic L-arginine supplementation, particularly in females (Figure 6D). No deaths occurred in young mice under control and L-arginine diet during the 4 months.

### Effects of Chronic L-Arginine Supplementation on Endothelial Dysfunction

**FIGURE 7:**
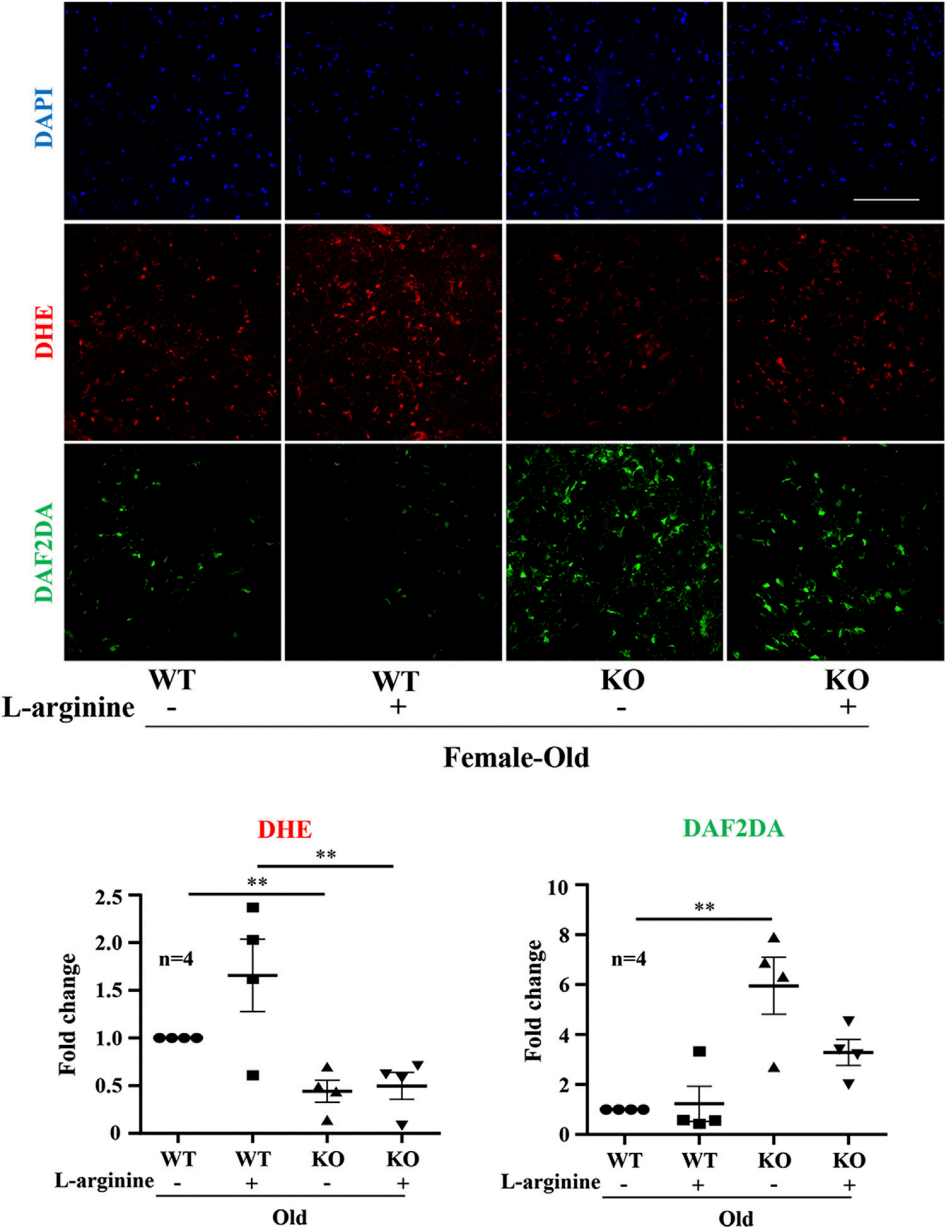
Long-term L-arginine supplementation causes vascular endothelial dysfunction. *En face* staining of aortic endothelial O_2_^−^ and NO production in old WT and Arg-II^−/−^ mice. Confocal microscopic *en face* detection of O_2_^−^ and NO by DHE and DAF-2DA staining, followed by counterstaining with DAPI of aortas. n = 4; **p < 0.01. Scale bar = 50 µm.

In the aortic endothelium, lower levels of superoxide anion (DHE staining) and higher levels of NO (DAF-2DA staining) were observed in the old Arg-II^−/−^ mice as compared to the WT animals (Figure 7). Importantly, L-arginine supplementation for 4 months increased superoxide anion production in the WT mice but not in the Arg-II^−/−^ mice. L-arginine supplementation had no effects on NO production in the WT mice. However, the increased NO levels in the Arg-II^−/−^ mice were reduced by L-arginine supplementation (Figure 7).

## Discussion

Studies with long-term L-arginine supplementation provide controversial results in animal experiments and also in clinical studies. Both beneficial and harmful effects are reported in the literature and stir continuous debates (Tome et al., 1999; Dioguardi, 2011; Nogiec and Kasif, 2013; Hadi et al., 2019; Nitz et al., 2019). At least two important randomized placebo-controlled clinical studies showed that 6-months L-arginine supplementation did not show any beneficial effects but harmful effects or increased mortality in patients with peripheral arterial disease and myocardial infarction on top of clinical medications (Schulman et al., 2006; Wilson et al., 2007). Despite the uncertain and controversial results, L-arginine supplementation is still considered as beneficial for health. There are substantial evidences showing that acute L-arginine supplementation is beneficial in terms of enhancing eNOS-derived NO production (Creager et al., 1992; Xiong et al., 2014) which is not necessarily resulting in sustained or long-term clinical outcome as above mentioned. In addition, high protein diet in which L-arginine is abundant is correlated with albumin hyperfiltration and chronic kidney damage (Brenner et al., 1982; O’Donnell et al., 1988; Juraschek et al., 2013). Therefore, we have designed the current study in mouse model to further analyze the effects of chronic L-arginine supplementation, focusing on age-associated kidney alterations.

Aging kidney is associated with inflammation and tubulointerstitial fibrosis (Hommos et al., 2017). Previous studies reported that L-arginine supplementation reduces inflammation and fibrosis in several nephropathy models (Morrissey et al., 1996; Peters et al., 2000; Kurus et al., 2005; Korish, 2010; Carlstrom et al., 2013). However, in our aging mouse model, we demonstrated that dietary L-arginine supplementation does not favorably affect but rather tends to further increase the aging kidney phenotypes, for example, for *il-1*, *icam-1*, *mcp1*, and the macrophage marker *f4/80*, *collagen Ia1*, *Ia2*, *and IIIa1*. The discrepancy of the effects of L-arginine could be due to the animal models used (disease vs. aging) or due to the long-term treatment of old mice, i.e., for 4 months as done in our study. More importantly, long-term L-arginine supplementation significantly enhanced albuminuria in the old mice and increased mortality during the 4 months of feeding which seems to be more pronounced in the female mice. The results of our study are in accordance with the findings reported by other studies showing increased albuminuria by L-arginine supplementation (Herlitz et al., 1999; Peters et al., 1999). This may be due to kidney damage and/or blockade of tubular protein reabsorption as reported in humans (Bello et al., 1999). Our findings on increased mortality rate in old animals by L-arginine supplementation are also seen in other animal models. For example, an increased mortality rate by L-arginine supplementation has been reported in experimental lupus nephritis and renal ischemia-injury mouse models (Tome et al., 1999; Peters et al., 2003). It is also in accordance with clinical data showing the higher mortality rate in patients with cardiovascular disease supplemented with 6 months L-arginine (Schulman et al., 2006). Moreover, the association of higher L-arginine level with increased risk of ischemic heart disease has also been demonstrated in humans (Au Yeung et al., 2016). The increased risk and mortality in patients or in aging models could be contributed by the detrimental effects of chronic L-arginine supplementation on kidney and also by eNOS dysfunction as shown by our present study and a clinical trial in patients with peripheral artery disease (Wilson et al., 2007). It is of note that dietary L-arginine supplementation has no significant effects on growth or body weight development in both young and old mice of males and females when compared to the mice without L-arginine supplementation, although it seems that body weight development is stimulated by L-arginine supplementation in the young female group. This may be due to the lean mass development under L-arginine feeding (McKnight et al., 2010). This effect does not occur in old mice whose anabolic metabolism is usually decreased.

The mechanisms of the harmful effects of chronic L-arginine supplementation remains unclear. There are *in vitro* experimental evidences in cultured human endothelial cells, demonstrating that exposure of the cells to L-arginine for a longer period of time suppresses eNOS expression and activity, induces oxidative stress, partly due to eNOS-uncoupling (Mohan et al., 2012; Xiong et al., 2014) caused by induction of the L-arginine metabolizing enzyme Arg-II and activation of S6K1 pathway (Scalera et al., 2009; Xiong et al., 2014), since Arg-II expression and S6K1 activation are induced by prolonged L-arginine exposure and genetic deficiency or silencing of Arg-II or S6K1 improves endothelial function in aging and under the L-arginine supplementation condition (Yepuri et al., 2012). In the present study with *in vivo* mouse model, we further confirmed that 4 months L-arginine supplementation enhances ROS generation in the old WT mice, which is prevented in Arg-II^−/−^. NO levels remain high in the Arg-II^−/−^ mice, although it tends to be reduced in these animals under L-arginine supplementation. It seems that Arg-II and S6K1 are not involved in increased albuminuria induced by chronic L-arginine supplementation. First, in the kidney, although we show an increase in S6K1 activity in aging, this is however, not significantly affected by L-arginine supplementation. Furthermore, we find a significantly increase in Arg-II in aging kidney, which is, also not significantly influenced by L-arginine supplementation. By using Arg-II^−/−^ mice, we observe that the L-arginine supplementation-induced increase in UACR is not affected by Arg-II deficiency, which implicates that the detrimental effects of long-term L-arginine supplementation are not mediated by S6K1 and Arg-II or Arg-II-derived metabolites. Since L-arginine is taken up by cationic amino acid transporters predominantly by CAT-2, and CAT-2 has been reported to be elevated in disease models including renal diseases (Schwartz et al., 2002; Schwartz et al., 2006; Huang et al., 2008) and deficiency of CAT-2 in mice showed beneficial effects in lung injury model, which seems to be partly due to reduced NO production derived from iNOS/L-arginine pathway (Jin et al., 2019). It remains to be investigated whether the detrimental effects of L-arginine supplementation on kidney UACR and mortality involves upregulation of CAT-2.

Another unanswered question is the gender difference of mice in response to chronic L-arginine supplementation. In general, females are protected by the sexual hormone estrogens and age-associated decline of estrogen levels contribute to cardiorenal disorders in elderly females (Gava et al., 2011). It is interesting to investigate the mechanisms of the more vulnerability of female mice to chronic L-arginine supplementation in terms of increased albuminuria and mortality under the regiment. There are studies showing regulatory effects of female hormones on L-arginine metabolism. It has been reported that estrogen increases L-arginine transporter activity in endothelial cells (Bentur et al., 2015) and reduces arginase expression (Hayashi et al., 2006). Future research shall investigate whether sex hormones could regulate transporters and enzymes involved in L-arginine uptake and metabolism and whether age-associated decline of estrogens could explain the gender biased detrimental effects of chronic L-arginine supplementation in females.

The results of our study do not show a direct evidence that increased death in females by chronic use of L-arginine is caused by kidney aging. It is also related to vascular endothelial dysfunction. Hence, we cautiously conclude that despite the limitations as above discussed, our study does not support the benefits of long-term L-arginine supplementation particularly in aging. It rather accelerates functional decline of kidney and vasculature in aging. The underlying mechanisms of detrimental effects of chronic L-arginine supplementation on vasculature and kidneys seem different. The former involves Arg-II, whereas the later seems not. Although many questions remain unanswered by this “clinical” study, taking the adverse effects of chronic L-arginine supplementation in patients and in this mouse model into account, the long-term dietary L-arginine supplementation should be used with caution or avoided particularly in elderly population/patients.

## Materials and Methods

### Materials

Reagents were purchased from the following sources: Mouse antibody against S6 (#2317s) and rabbit antibodies against phospho S6-S240/244 (#5364) and Arg-II (#55003S) were purchased from Cell Signaling Technology; Mouse antibody against Tubulin (T5168) and Goat Serum Donor Herd (G6767) were from MilliporeSigma (Burlington, MA, United States); Mouse antibody against p16 (sc-81156) was purchased from Santa Cruz Technology Inc. (Dallas, United States); Secondary antibodies IRDye 800-conjugated affinity purified goat anti-rabbit IgG was purchased from BioConcept (Alschwil, Switzerland) and Alexa fluor 680-conjugated goat anti-mouse IgG was from Invitrogen (Lucerne, Switzerland); Mouse IgG blocking reagent (MKB-2213) was from Vector Laboratories (Peterborough, United Kingdom); Alexa Fluor 488 conjugated-Goat anti-mouse IgG(H + L) secondary antibody (A-11001) was from Thermo Fisher Scientific (Waltham, MA United States); Dihydroethidium (DHE) was from Molecular Probes/Invitrogen (Lucerne, Switzerland), and the membrane-permeable 4,5-diaminofluoresceine diacetate (DAF-2DA) was from VWR international SA (Dietikon, Switzerland).

### Animals

Arg-II^−/−^ mice were kindly provided by Dr William O’Brien (Shi et al., 2001) and backcrossed to C57BL/6J for more than 10 generations. Genotyping was performed by polymerase chain reaction (PCR) as previously described (Shi et al., 2001). Wild-type (WT) and Arg-II^−/−^ offspring from hetero/hetero cross were interbred to obtain WT and Arg-II^−/−^ mice, respectively, for experiments. Mice were housed at 23°C, with 12 h light-dark cycle, and fed a normal chow diet with free access to tap water. Starting at the age of 4 months (young group) and 18–24 months (old group), male and female WT and Arg-II^−/−^ mice were given free access to water and fed with a standard chow (0.65% L-arginine) or L-arginine supplemented chow (0.65% + 1,815%) (Table 1) during 16 weeks, and maintained on a 12 h light-dark cycle according to the local guidelines of animal experimentation. Animals were controlled every 2-weeks for body weight measurements. Before being euthanized, animals were starved for 12 h, and after, kidney tissue was collected by snap freezing into liquid nitrogen. Animal work was approved by the Ethical Committee of Veterinary Office of Fribourg Switzerland (2013_08_FR and 2016_43_FR) and performed in compliance with guidelines on animal experimentation at our institution.

**TABLE 1:** Composition of control and L-Arginine rich food in this study.

Component	Control	L-arginine rich food
	%	%
	2222 Kliba	2222 modified with L-Arginine
Zucker	11.86	11.86
Choline	0.27	0.27
Dextrose	13.2	13.2
Maisstaerke	39.77	39.77
Cystine-L	0.33	0.33
Sojaoel Raffieniert	7	7
BHT	0.01	0.01
Casein (Reagent Grade 90%)	**20**	**18**
Cellulose	5	5.05
VIT MIX AIN93G	0.2	0.2
VM Spuren MIX AIN 93G	0.3	0.3
Arginine from Sigma (A5006, Grade > 98%)		**1.8**
Total	**97.94**	**95.99**
% Protein	**18.00**	**18**
% L-Arginine from casein	**0.65**	**0.585**
% Supplememted L-Arginine		**1.88**
Final Arginine content	**0.65**	**2.46**
ME kcal/kg	3690	3690
% ME from protein	19.5	19.5
% ME from carbohydrates	63.4	63.4
% ME from fat	17.1	17.1

The food is from KLIBA.. Control diet is 2222 Kliba, 2222 modified with arginine differs from 2222 Kliba in the amount of casein and L-arginine. In comparison to 2222 Kliba, it contains 18% instead of 20% of casein and 2.46% instead of 0.65% of L-arginine. Bold values highlight the difference between the two types of food.

### Metabolic Cage Experiments

Before metabolic cage experiments (Indulab, Gams, Switzerland), mice were acclimatized individually for 3 days (8 h/d in metabolic cage). After acclimation, metabolic cage experiments were performed on each mouse at night-time period (active phase) from 7:00 PM to 7:00 AM. During experiments, mice had free access to water and were food deprived, to avoid any food contamination in collected urine. Water intake was measured and urine was collected.

### Measurement of Blood Urea Nitrogen, Creatinine, and Albuminuria

Urea nitrogen concentrations in plasma samples were measured using commercially available spectrophotometric assays (Urea Nitrogen Colorimetric Detection Kit, K024-H1, Arbor Assays, Ann Arbor, United States) according to the manufacturer’s instruction (Li et al., 2019). Briefly, samples were diluted 1:25 with distilled water and 50 µl of diluted samples and standards were mixed with Color Reagent A and Color Reagent B. Absorbance was measured at 450 nm after incubation for 30 min.

Measurement of urine creatinine was kindly performed by Laboratory HFR, Hospital Fribourgeois using the Jaffe’s method. Albuminuria was measured by ELISA using a mouse Albuwell kit (1,011**,** Exocell Inc., Philadelphia, PA, United States), according to the manufacturer’s instructions (Beckerman et al., 2017). Briefly, urine samples diluted 1:25 with NHEBSA and 50 µl of diluted samples and standards were incubated with 30 µl of anti-mouse Albumin Ab-HRP conjugate for 30 min. Subsequently, 100 µl of Color Developer was added to each well and incubated for 10 min. The reaction was stopped by adding 100 µl of Color Stopper. Absorbance was measured at 450 nm. Albumin was normalized by creatinine and albuminuria was quantified by albumin/creatinine ratio (UACR, μg/mg). Both creatinine and albumin were measured from 12 h night-time urine collected from animals in metabolic cages.

### Western Blotting

Tissue lysate preparation, SDS-PAGE and immunoblotting, antibody incubation and signal detection were performed as described previously Ming et al. (2012). In brief, frozen kidney was ground to a fine powder using a mortar and pestle in a liquid nitrogen bath. A portion of fine powder was then homogenized in 150 µl of ice-cold lysis buffer (20 mM Tris [pH 8.0], 138 mM NaCl, 2.7 µM KCl, 1 µM MgCl, 1 mM CaCl_2_, 1 mM NaVO_4_, 20 mM NaF, 5 mM EDTA, 5% glycerol, 1% NP-40, protease inhibitor cocktail (B14002 Biotool, Munich, Germany) and phosphatase inhibitor cocktail (B15002; Biotool, Munich, Germany) with XENOX-Motorhandstück MHX homogenizer on ice. Homogenate was centrifuged in a Sorvall Legend Micro 17R at 13,800 g for 15 min at 4°C, and protein concentration of the supernatant was determined by the Lowry method (500-0116, Bio-Rad). Lysates that contained equal amounts of protein were heated at 75°C for 15 min in Laemmli buffer and separated by 10% SDS-PAGE, then transferred to PVDF membranes. The resultant membranes were blocked with PBS-Tween 20 that was supplemented with 5% nonfat dry milk, then incubated with the corresponding primary antibody overnight at 4°C with gentle agitation. After washing, the blot was then further incubated with corresponding anti-mouse (Alexa Fluor 680-conjugated) or anti-rabbit (IRDye 800-conjugated) secondary antibodies. Signals were visualized using the Odyssey Infrared Imaging System (LI-COR Biosciences, United States). Quantification of the signals was performed using NIH Image 1.60 software (US National Institutes of Health).

### Real-Time Quantitative Real Time-PCR

mRNA expression of the several inflammatory markers, collagens, *p16* ^*INK4a*^ and Ribosomal Protein S12 (*RPS12*) was measured by two-step quantitative Real Time-PCR as described previously Ming et al. (2012). Total RNA was extracted from kidney with Trizol Reagent (TR-118, Molecular Research Center, Inc., Cincinnati, OH, United States) following manufacturer’s protocol. Real-time PCR reaction was performed with the GoTaq® qPCR Master Mix (A6001, Promega) and iCycler system (Bio-Rad). The mRNA expression levels of all genes was quantified using the standard curve method and were further normalized to the reference gene *RPS12*. The following primer sequences of mouse origin were used:

*collagen Iα1*-F: 5′-TGG CCA AGA AGA CAT CCC TGA AGT C-3′

*collagen Iα1*-R: 5′-GGC AGA TAC AGA TCA AGC ATA CCT CGG-3′

*collagen Iα2*-F: 5′-CTG GTC TTA CTG GGA ACT TTG CTG C-3′

*collagen Iα2*-R: 5′-CCA ACA GCA CCA GGA GGG CC-3′

*collagen IIIα1*-F: 5′-CAA ACA CGC AAG GCA ATG AGA CTA CC-3′

*collagen IIIα1*-R: 5′-AGG GCC AAT GTC CAC ACC AAA TTC-3′

*f4/80*-F: 5′-TGG CTG CCT CCC TGA CTT TC-3′

*f4/80*-R: 5′-CAA GAT CCC TGC CCT GCA CT-3′

*icam-1*-F: 5′-TGC TTT GAG AAC TGT GGC AC-3′

*icam-1*-R: 5′-GCT CAG TAT CTC CTC CCC AC-3′

*il6*-F: 5′-GAC AAC CAC GGC CTT CCC TA-3′

*il6*-R: 5′-GCC TCC GAC TTG TGA AGT GGT-3′

*il-1β* -F: 5′-GCA ACT GTT CCT GAA CTC AAC T-3′

*il-1β*-R: 5′-TCT TTT GGG GTC CGT CAA CT-3′

*inos*-F: 5′-GGC AAA CCC AAG GTC TAC GTT-3′

*inos*-R: 5′-TCG CTC AAG TTC AGC TTG GT-3′

*mcp1*-F: 5′-AGC ACC AGC CAA CTC TCA C-3′

*mcp1*-R: 5′-TCT GGA CCC ATT CCT TCT TG-3′

*p16* ^*INK4a*^-F: 5′-GAA CTC TTT CGG TCG TAC-3′

*p16* ^*INK4a*^-R: 5′GCA GAA GAG CTG CTA CGT-3′

*rps12*-F: 5′-GAA GCT GCC AAA GCC TTA GA-3′

*rps12*-R: 5′-AAC TGC AAC CAA CCA CCT TC-3′

*tnf-α*-F: 5′-GGC AGG TCT ACT TTG GAG TCA TTG C-3′

*tnf-α*-R: 5′-ACA TTC GAG GCT CCA GTG AAT TCG G-3′

*vcam-1*-F: 5′-ACA GAC AGT CCC CTC AAT GG-3′

*vcam-1*-R: 5′-ACA GTG ACA GGT CTC CCA TG-3′

### Immunofluorescence Staining

Kidneys were isolated and fixed with 10% Neutral buffered formalin (NBF) and embedded in paraffin. After deparaffinization in xylene (3 times, 5 min for each), hydration in ethanol (twice in 100% ethanol, twice in 95% ethanol, and once in 80%, 75%, 50% ethanol for 5 min, sequentially), heat-induced epitope retrieval in Citrate buffer (10 mM Citric Acid, 0.05% Tween-20, pH 6.0) was performed at 95–100°C for 30 min to unmask antigens present in renal tissue. Tissue sections (5 μm) were then blocked with mouse IgG blocking reagent for 3 h and with PBS that contained 1% BSA and 10% goat serum for 1 h, sequentially. Then sections were incubated with mouse anti-P16 antibody (1:50) at 4°C overnight and subsequently with Alexa Fluor 488–conjugated goat anti-Mouse IgG (H + L) Highly Cross-Adsorbed Secondary antibody for 2 h at room temperature, followed by counterstaining with DAPI. Negative control was performed by using IgG instead of primary antibody. Immunofluorescence signals were visualized under Leica DM6B Navigator. The intensity of the fluorescence was quantified by Leica Application Suite X (LAS X) software.

### En Face Detection of Superoxide Anion and Nitric Oxide in Mouse Aortas

Superoxide anoin and NO production was assessed with DHE and DAF-2DA staining as described previously Yu et al. (2016). Briefly, old female mice aortas cleaned of perivascular tissues were equilibrated for 30 min in Krebs buffer at 37°C aerated with 95% O_2_ and 5% CO_2_. DHE/DAF-2DA (5 μmol/L of each dye) was then added for 30 min. The aortas were then washed three times and fixed in 4% paraformaldehyde followed by counterstaining with DAPI (300 nmol/L for 3 min). After washing with phosphate buffered saline (PBS), the aortas were carefully cut longitudinally and mounted en face (face down) on slides and then covered with cover slip for endothelial layer imaging. The images from DHE, DAF-2DA, and DAPI staining were quantified with Image J software and results are presented as the ratio of DAF-2DA and DAPI positive nucleus or ratio of DHE and DAPI.

### Statistics

Statistical analysis was performed with unpaired Student’s *t*-Test or analysis of variance (ANOVA) with Tukey-test. Kolmogorov-Smirnov test was used to determine if data is normally distributed. Gehan-Breslow-Wilcoxon test was used for the survival curves analysis. Statistical differences in mean values were considered significant at two tailed *p* ≤ 0.05. Data are given as mean ± SEM and n represents the number of animals for each group.

## Data Availability Statement

The raw data supporting the conclusions of this article will be made available by the authors, without undue reservation, to any qualified researcher.

## Ethics Statement

The animal study was reviewed and approved by Ethical Committee of Veterinary Office of Fribourg Switzerland (2013_08_FR and 2016_43_FR).

## Author Contributions

JH and DL: Performance of experiments, collection and analysis of data, interpretation of data, manuscript writing; YY: Performance of experiments, collection, analysis, and interpretation of the data on blood vessels; X-FM and ZY: Conception and design of the project, financial support of the project, data analysis and interpretation of data, and manuscript writing and approval of the manuscript.

## Funding

This work was supported by the Swiss National Science Foundation (31003A_159582/1 and 31003A_179261/1), Swiss Heart Foundation (FF19033), and National Centre of Competence in Research Program (NCCR-Kidney.CH).

## Conflict of Interest

The authors declare that the research was conducted in the absence of any commercial or financial relationships that could be construed as a potential conflict of interest.

## References

[B1] Au Yeung S. L. Lin S. L. Lam H. S. Schooling C. M. (2016). Effect of l-arginine, asymmetric dimethylarginine, and symmetric dimethylarginine on ischemic heart disease risk: a Mendelian randomization study. Am. Heart J. 182, 54–61. 10.1016/j.ahj.2016.07.021 27914500

[B2] Baylis C. (2006). Arginine, arginine analogs and nitric oxide production in chronic kidney disease. Nat. Clin. Pract. Nephrol. 2, 209–220. 10.1038/ncpneph0143 16932427 PMC2756810

[B3] Beckerman P. Bi-Karchin J. Park A. S. Qiu C. Dummer P. D. Soomro I. (2017). Transgenic expression of human APOL1 risk variants in podocytes induces kidney disease in mice. Nat. Med. 23, 429–438. 10.1038/nm.4287 28218918 PMC5603285

[B4] Bello E. Caramelo C. Lopez M. D. Soldevilla M. J. Gonzalez-Pacheco F. R. Rovira A. (1999). Induction of microalbuminuria by l-arginine infusion in healthy individuals: an insight into the mechanisms of proteinuria. Am. J. Kidney Dis. 33, 1018–1025. 10.1016/S0272-6386(99)70137-X 10352188

[B5] Bentur O. S. Schwartz D. Chernichovski T. Ingbir M. Weinstein T. Chernin G. (2015). Estradiol augments while progesterone inhibits arginine transport in human endothelial cells through modulation of cationic amino acid transporter-1. Am. J. Physiol. Regul. Integr. Comp. Physiol. 309, R421–R427. 10.1152/ajpregu.00532.2014 26062636

[B6] Brenner B. M. Meyer T. W. Hostetter T. H. (1982). Dietary protein intake and the progressive nature of kidney disease: the role of hemodynamically mediated glomerular injury in the pathogenesis of progressive glomerular sclerosis in aging, renal ablation, and intrinsic renal disease. N. Engl. J. Med. 307, 652–659. 10.1056/NEJM198209093071104 7050706

[B7] Carlstrom M. Brown R. D. Yang T. Hezel M. Larsson E. Scheffer P. G. (2013). L-arginine or tempol supplementation improves renal and cardiovascular function in rats with reduced renal mass and chronic high salt intake. Acta Physiol. 207, 732–741. 10.1111/apha.12079 23387940

[B8] Chen J. Kuhlencordt P. Urano F. Ichinose H. Astern J. Huang P. L. (2003). Effects of chronic treatment with L-arginine on atherosclerosis in apoE knockout and apoE/inducible NO synthase double-knockout mice. Arterioscler. Thromb. Vasc. Biol. 23, 97–103. 10.1161/01.atv.0000040223.74255.5a 12524231

[B9] Creager M. A. Gallagher S. J. Girerd X. J. Coleman S. M. Dzau V. J. Cooke J. P. (1992). L-arginine improves endothelium-dependent vasodilation in hypercholesterolemic humans. J. Clin. Invest. 90, 1248–1253. 10.1172/JCI115987 1401062 PMC443166

[B10] Dioguardi F. S. (2011). To give or not to give? Lessons from the arginine paradox. J.Nutrigenet. Nutrigenomics 4, 90–98. 10.1159/000327777 21625171

[B11] Donato A. J. Machin D. R. Lesniewski L. A. (2018). Mechanisms of dysfunction in the aging vasculature and role in age-related disease. Circ. Res. 123, 825–848. 10.1161/CIRCRESAHA.118.312563 30355078 PMC6207260

[B12] Dong J. Y. Qin L. Q. Zhang Z. Zhao Y. Wang J. Arigoni F. (2011). Effect of oral L-arginine supplementation on blood pressure: a meta-analysis of randomized, double-blind, placebo-controlled trials. Am. Heart J. 162, 959–965. 10.1016/j.ahj.2011.09.012 22137067

[B13] Friedman A. N. (2004). High-protein diets: potential effects on the kidney in renal health and disease. Am. J. Kidney Dis. 44, 950–962. 10.1053/j.ajkd.2004.08.020 15558517

[B14] Gava A. L. Freitas F. P. Meyrelles S. S. Silva I. V. Graceli J. B. (2011). Gender-dependent effects of aging on the kidney. Braz. J. Med. Biol. Res. 44, 905–913. 10.1590/s0100-879x2011007500101 21956533

[B15] Hadi A. Arab A. Moradi S. Pantovic A. Clark C. C. T. Ghaedi E. (2019). The effect of l-arginine supplementation on lipid profile: a systematic review and meta-analysis of randomised controlled trials. Br. J. Nutr. 122, 1021–1032. 10.1017/S0007114519001855 31922465

[B16] Hayashi T. Esaki T. Sumi D. Mukherjee T. Iguchi A. Chaudhuri G. (2006). Modulating role of estradiol on arginase II expression in hyperlipidemic rabbits as an atheroprotective mechanism. Proc. Natl. Acad. Sci. U.S.A. 103, 10485–10490. 10.1073/pnas.0603918103 16801563 PMC1502484

[B17] Herlitz H. Jungersten L. U. Wikstrand J. Widgren B. R. (1999). Effect of L-arginine infusion in normotensive subjects with and without a family history of hypertension. Kidney Int. 56, 1838–1845. 10.1046/j.1523-1755.1999.00735.x 10571792

[B18] Hommos M. S. Glassock R. J. Rule A. D. (2017). Structural and functional changes in human kidneys with healthy aging. J. Am. Soc. Nephrol. 28, 2838–2844. 10.1681/ASN.2017040421 28790143 PMC5619977

[B19] Huang J. Rajapakse A. Xiong Y. Montani J.-P. Verrey F. Ming X.-F. (2016). Genetic targeting of arginase-II in mouse prevents renal oxidative stress and inflammation in diet-induced obesity. Front. Physiol. 7, 560 10.3389/fphys.2016.00560 27920727 PMC5118905

[B20] Huang T. Y. Tsai P. S. Huang C. J. (2008). HO-1 overexpression attenuates endotoxin effects on CAT-2 isozymes expression. J. Surg. Res. 148, 172–180. 10.1016/j.jss.2007.06.027 18028947

[B21] Jin Y. Liu Y. Nelin L. D. (2019). Deficiency of cationic amino acid transporter-2 protects mice from hyperoxia-induced lung injury. Am. J. Physiol. Lung Cell Mol. Physiol. 316, L598–L607. 10.1152/ajplung.00223.2018 30628488 PMC6842872

[B22] Juraschek S. P. Appel L. J. Anderson C. A. Miller E. R. 3rd (2013). Effect of a high-protein diet on kidney function in healthy adults: results from the OmniHeart trial. Am. J. Kidney Dis. 61, 547–554. 10.1053/j.ajkd.2012.10.017 23219108 PMC3602135

[B23] Korish A. A. (2010). Multiple antioxidants and L-arginine modulate inflammation and dyslipidemia in chronic renal failure rats. Ren. Fail. 32, 203–213. 10.3109/08860221003592820 20199183

[B24] Kurus M. Esrefoglu M. Bay A. Ozturk F. (2005). Protective effect of oral L-arginine supplementation on cyclosporine induced nephropathy in rats. Int. Urol. Nephrol. 37, 587–594. 10.1007/s11255-004-0011-5 16307347

[B25] Li Y. Hu Q. Li C. Liang K. Xiang Y. Hsiao H. (2019). PTEN-induced partial epithelial-mesenchymal transition drives diabetic kidney disease. J. Clin. Invest. 129, 1129–1151. 10.1172/JCI121987 30741721 PMC6391108

[B26] Makrides V. Camargo S. M. Verrey F. (2014). Transport of amino acids in the kidney. Compr. Physiol. 4, 367–403. 10.1002/cphy.c130028 24692143

[B27] Maxwell A. J. Anderson B. Zapien M. P. Cooke J. P. (2000). Endothelial dysfunction in hypercholesterolemia is reversed by a nutritional product designed to enhance nitric oxide activity. Cardiovasc. Drugs Ther. 14, 309–316. 10.1023/a:1007886725480 10935153

[B28] Mcknight J. R. Satterfield M. C. Jobgen W. S. Smith S. B. Spencer T. E. Meininger C. J. (2010). Beneficial effects of L-arginine on reducing obesity: potential mechanisms and important implications for human health. Amino Acids 39, 349–357. 10.1007/s00726-010-0598-z 20437186

[B29] Meirelles C. M. Matsuura C. (2018). Acute supplementation of L-arginine affects neither strength performance nor nitric oxide production. J. Sports Med. Phys. Fitness 58, 216–220. 10.23736/S0022-4707.16.06680-9 27623757

[B30] Ming X. F. Rajapakse A. G. Yepuri G. Xiong Y. Carvas J. M. Ruffieux J. (2012). Arginase II promotes macrophage inflammatory responses through mitochondrial reactive oxygen species, contributing to insulin resistance and atherogenesis. J. Am. Heart Assoc. 1, e000992 10.1161/JAHA.112.000992 23130157 PMC3487353

[B31] Mohan S. Wu C. C. Shin S. Fung H. L. (2012). Continuous exposure to L-arginine induces oxidative stress and physiological tolerance in cultured human endothelial cells. Amino Acids 43, 1179–1188. 10.1007/s00726-011-1173-y 22130739 PMC3321093

[B32] Moretto J. Girard C. Demougeot C. (2019). The role of arginase in aging: a systematic review. Exp. Gerontol. 116, 54–73. 10.1016/j.exger.2018.12.011 30578842

[B33] Morrissey J. J. Ishidoya S. Mccracken R. Klahr S. (1996). Nitric oxide generation ameliorates the tubulointerstitial fibrosis of obstructive nephropathy. J. Am. Soc. Nephrol. 7, 2202–2212. 8915981 10.1681/ASN.V7102202

[B34] Nitz K. Lacy M. Atzler D. (2019). Amino acids and their metabolism in atherosclerosis. Arterioscler. Thromb. Vasc. Biol. 39, 319–330. 10.1161/ATVBAHA.118.311572 30650999

[B35] Nogiec C. D. Kasif S. (2013). To supplement or not to supplement: a metabolic network framework for human nutritional supplements. PLoS One 8, e68751 10.1371/journal.pone.0068751 23967053 PMC3740736

[B36] O’donnell M. P. Kasiske B. L. Keane W. F. (1988). Glomerular hemodynamic and structural alterations in experimental diabetes mellitus. FASEB J. 2, 2339–2347. 10.1096/fasebj.2.8.3282959 3282959

[B37] O'sullivan E. D. Hughes J. Ferenbach D. A. (2017). Renal aging: causes and consequences. J. Am. Soc. Nephrol. 28, 407–420. 10.1681/ASN.2015121308 28143966 PMC5280008

[B38] Peters H. Border W. A. Noble N. A. (1999). L-Arginine supplementation increases mesangial cell injury and subsequent tissue fibrosis in experimental glomerulonephritis. Kidney Int. 55, 2264–2273. 10.1046/j.1523-1755.1999.00462.x 10354274

[B39] Peters H. Border W. A. Noble N. A. (2000). Tandem antifibrotic actions of L-arginine supplementation and low protein diet during the repair phase of experimental glomerulonephritis. Kidney Int. 57, 992–1001. 10.1046/j.1523-1755.2000.00927.x 10720952

[B40] Peters H. Border W. A. Ruckert M. Kramer S. Neumayer H. H. Noble N. A. (2003). L-arginine supplementation accelerates renal fibrosis and shortens life span in experimental lupus nephritis. Kidney Int. 63, 1382–1392. 10.1046/j.1523-1755.2003.00881.x 12631354

[B41] Rector T. S. Bank A. J. Mullen K. A. Tschumperlin L. K. Sih R. Pillai K. (1996). Randomized, double-blind, placebo-controlled study of supplemental oral L-arginine in patients with heart failure. Circulation 93, 2135–2141. 10.1161/01.cir.93.12.2135 8925582

[B42] Rodrigues-Krause J. Krause M. Rocha I. Umpierre D. Fayh A. P. T. (2018). Association of l-arginine supplementation with markers of e ndothelial function in patients with cardiovascular or metabolic disorders: a systematic review and meta-analysis. Nutrients 11, 15 10.3390/nu11010015 PMC6357192 30577559

[B43] Sato K. Kinoshita M. Kojima M. Miyagawa K. Takase H. Suzuki S. (2000). Failure of L-arginine to induce hypotension in patients with a history of accelerated-malignant hypertension. J. Hum. Hypertens. 14, 485–488. 10.1038/sj.jhh.1001064 10962515

[B44] Scalera F. Closs E. I. Flick E. Martens-Lobenhoffer J. Boissel J. P. Lendeckel U. (2009). Paradoxical effect of L-arginine: acceleration of endothelial cell senescence. Biochem. Biophys. Res. Commun. 386, 650–655. 10.1016/j.bbrc.2009.06.091 19545540

[B45] Schmitt R. Melk A. (2017). Molecular mechanisms of renal aging. Kidney Int. 92, 569–579. 10.1016/j.kint.2017.02.036 28729036

[B46] Schulman S. P. Becker L. C. Kass D. A. Champion H. C. Terrin M. L. Forman S. (2006). L-arginine therapy in acute myocardial infarction: the Vascular Interaction with Age in Myocardial Infarction (VINTAGE MI) randomized clinical trial. J. Am. Med. Assoc. 295, 58–64. 10.1001/jama.295.1.58 16391217

[B47] Schwartz I. F. Chernichovsky T. Hagin D. Ingbir M. Reshef R. Chernin G. (2006). Differential regulation of L-arginine transporters (cationic amino acid transporter-1 and -2) by peroxynitrite in rat mesangial cells. Nephrol. Dial. Transplant. 21, 3409–3414. 10.1093/ndt/gfl522 16998217

[B48] Schwartz I. F. Schwartz D. Traskonov M. Chernichovsky T. Wollman Y. Gnessin E. (2002). L-Arginine transport is augmented through up-regulation of tubular CAT-2 mRNA in ischemic acute renal failure in rats. Kidney Int. 62, 1700–1706. 10.1046/j.1523-1755.2002.t01-1-00622.x 12371970

[B49] Shi O. Morris S. M. Jr. Zoghbi H. Porter C. W. O’brien W. E. (2001). Generation of a mouse model for arginase II deficiency by targeted disruption of the arginase II gene. Mol. Cell. Biol. 21, 811–813. 10.1128/MCB.21.3.811-813.2001 11154268 PMC86672

[B50] Tome L. A. Yu L. De Castro I. Campos S. B. Seguro A. C. (1999). Beneficial and harmful effects of L-arginine on renal ischaemia. Nephrol. Dial. Transplant. 14, 1139–1145. 10.1093/ndt/14.5.1139 10344352

[B51] Wilson A. M. Harada R. Nair N. Balasubramanian N. Cooke J. P. (2007). L-arginine supplementation in peripheral arterial disease: no benefit and possible harm. Circulation 116, 188–195. 10.1161/CIRCULATIONAHA.106.683656 17592080

[B52] Wu G. Bazer F. W. Davis T. A. Kim S. W. Li P. Marc Rhoads J. (2009). Arginine metabolism and nutrition in growth, health and disease. Amino Acids 37, 153–168. 10.1007/s00726-008-0210-y 19030957 PMC2677116

[B53] Xiong Y. Fru M. F. Yu Y. Montani J. P. Ming X. F. Yang Z. (2014). Long term exposure to L-arginine accelerates endothelial cell senescence through arginase-II and S6K1 signaling. Aging (Albany NY) 6, 369–379. 10.18632/aging.100663 24860943 PMC4069264

[B54] Yepuri G. Velagapudi S. Xiong Y. Y. Rajapakse A. G. Montani J. P. Ming X. F. (2012). Positive crosstalk between arginase-II and S6K1 in vascular endothelial inflammation and aging. Aging Cell 11, 1005–1016. 10.1111/Acel.12001 22928666

[B55] You H. Gao T. Cooper T. K. Morris S. M. Jr. Awad A. S. (2014). Diabetic nephropathy is resistant to oral L-arginine or L-citrulline supplementation. Am. J. Physiol. Renal. Physiol. 307, F1292–F1301. 10.1152/ajprenal.00176.2014 25320354 PMC4254967

[B56] Yu Y. Xiong Y. Montani J. P. Yang Z. Ming X. F. (2016). En face detection of nitric oxide and superoxide in e ndothelial layer of intact arteries. J. Vis. Exp. 25, 53718 10.3791/53718 PMC4828200 26967197

