# Supplementary material for: Detrimental Effects of Chronic L-Arginine Rich Food on Aging Kidney
Source: Front Pharmacol. 2021 Jan 19;11:582155. doi: 10.3389/fphar.2020.582155 (PMC7851093; doi:10.3389/fphar.2020.582155)
Supplement: Supplementary file 1 [file presentation1.pptx]

## Slide 1
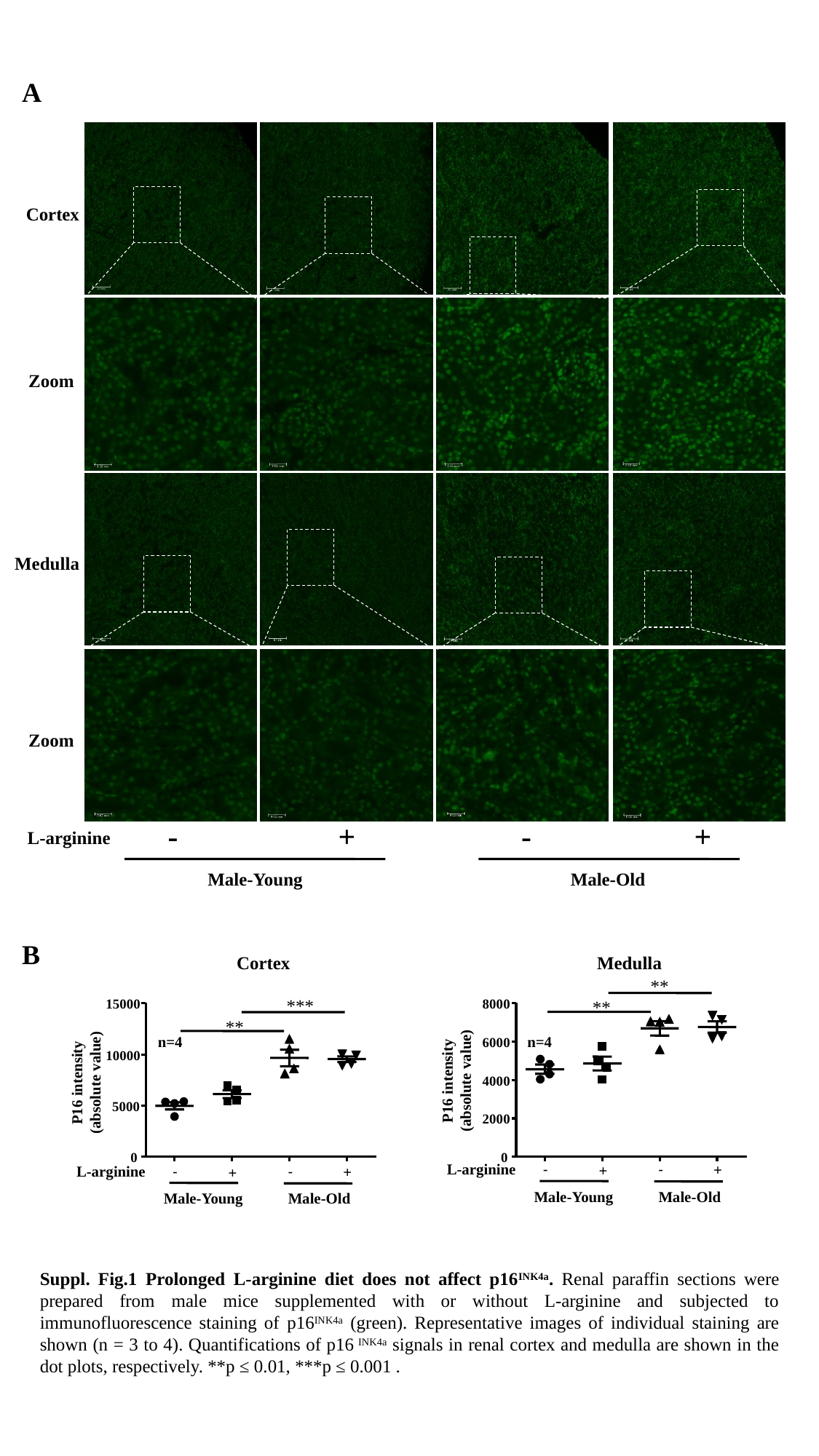

A
Cortex
Zoom
Medulla
Zoom
-
+
-
+
L-arginine
Male-Young
Male-Old
B
 Medulla
 Cortex
**
***
15000
8000
**
**
n=4
n=4
6000
10000
P16 intensity
(absolute value)
P16 intensity
(absolute value)
4000
5000
2000
0
0
L-arginine
-
-
+
+
L-arginine
-
-
+
+
Male-Young
Male-Old
Male-Young
Male-Old
Suppl. Fig.1 Prolonged L-arginine diet does not affect p16INK4a. Renal paraffin sections were prepared from male mice supplemented with or without L-arginine and subjected to immunofluorescence staining of p16INK4a (green). Representative images of individual staining are shown (n = 3 to 4). Quantifications of p16 INK4a signals in renal cortex and medulla are shown in the dot plots, respectively. **p ≤ 0.01, ***p ≤ 0.001 .
