# Supplementary material for: Detrimental Effects of Chronic L-Arginine Rich Food on Aging Kidney
Source: Front Pharmacol. 2021 Jan 19;11:582155. doi: 10.3389/fphar.2020.582155 (PMC7851093; doi:10.3389/fphar.2020.582155)
Supplement: Supplementary file 2 [file presentation2.pptx]

## Slide 1
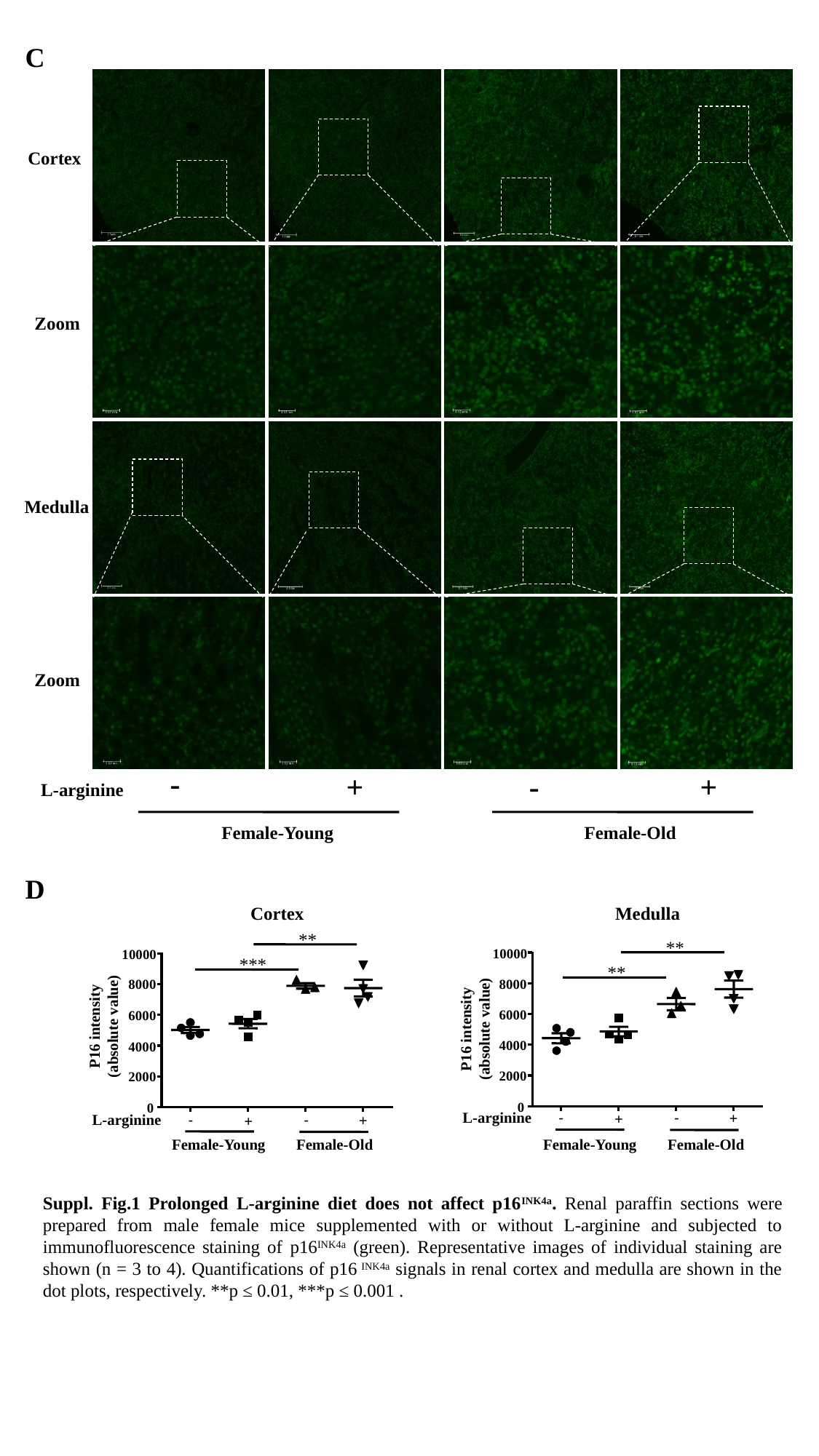

C
Cortex
Zoom
Medulla
Zoom
-
+
+
-
L-arginine
Female-Young
Female-Old
D
 Medulla
 Cortex
**
**
10000
10000
***
**
8000
8000
P16 intensity
(absolute value)
P16 intensity
(absolute value)
6000
6000
4000
4000
2000
2000
0
0
L-arginine
-
-
+
+
L-arginine
-
-
+
+
Female-Young
Female-Old
Female-Young
Female-Old
Suppl. Fig.1 Prolonged L-arginine diet does not affect p16INK4a. Renal paraffin sections were prepared from male female mice supplemented with or without L-arginine and subjected to immunofluorescence staining of p16INK4a (green). Representative images of individual staining are shown (n = 3 to 4). Quantifications of p16 INK4a signals in renal cortex and medulla are shown in the dot plots, respectively. **p ≤ 0.01, ***p ≤ 0.001 .
